# Prescription drug monitoring program use by opioid prescribers: a cross-sectional study

**DOI:** 10.1093/haschl/qxad067

**Published:** 2023-11-30

**Authors:** Adam Sacarny, Ian Williamson, Weston Merrick, Tatyana Avilova, Mireille Jacobson

**Affiliations:** Department of Health Policy and Management, Mailman School of Public Health, Columbia University, New York, NY 10032, United States; National Bureau of Economic Research, Cambridge, MA 02138, United States; Abdul Latif Jameel Poverty Action Lab (J-PAL), Cambridge, MA 02142, United States; Minnesota Management and Budget Agency, Saint Paul, MN 55155, United States; Minnesota Management and Budget Agency, Saint Paul, MN 55155, United States; Department of Economics, Bowdoin College, Brunswick, ME 04011, United States; National Bureau of Economic Research, Cambridge, MA 02138, United States; The Leonard Davis School of Gerontology, Los Angeles, CA 90089, United States; The Schaeffer Center for Health Policy & Economics, Los Angeles, CA 90089, United States

**Keywords:** prescription drug monitoring programs, opioids, prescribing, controlled substances

## Abstract

Clinician use of prescription drug monitoring programs (PDMPs) has been linked to better patient outcomes, but state requirements to use PDMPs are unevenly enforced. We assessed PDMP use in Minnesota, which requires opioid prescribers to hold accounts and, in most cases, search the PDMP before prescribing, but where enforcement authority is limited. Using 2023 PDMP data, we found that 4 in 10 opioid prescribers did not search and 2 in 10 did not hold an account. PDMP use was strongly associated with prescribing volume, but even among the top decile of opioid prescribers, 8% never searched the PDMP. Thirty-two percent of opioid fills came from clinicians who did not search the PDMP. Failures to use the PDMP may be driven by a lack of information about state requirements, beliefs that these requirements are not enforced, and the costs of accessing the PDMP relative to the benefits. These results highlight the potential for policy makers to promote safer and better-informed prescribing of opioids and other drugs by addressing the forces that have limited PDMP use so far.

## Introduction

Prescription drug monitoring programs (PDMPs), databases of controlled-substance dispensing that clinicians can query to monitor patient risk for overdose and other adverse events, currently operate in all states. Despite their ubiquity, research suggests that the benefits of PDMPs alone for patient outcomes are modest.^1^ Mandates that require PDMP use have shown more promise, suggesting that clinician engagement is key to the success of these programs.^2,3^ Consequently, most states now require that clinicians have PDMP accounts and check the database before prescribing, but these rules are unevenly enforced.^4,5^ We examined PDMP use among opioid prescribers in Minnesota, where all controlled-substance prescribers are required to make accounts and search the PDMP before writing most opioid prescriptions but where administrators have limited authority to enforce this mandate.

## Data and methods

We obtained data on the universe of Minnesota controlled-substance prescribing, PDMP searches, and PDMP accounts from the Minnesota Prescription Monitoring Program. We then analyzed PDMP search and account-holding by prescriber characteristics for the 180-day period starting February 1, 2023. To identify Minnesota-based prescribers, we used National Plan and Provider Enumeration System data. We identified opioid prescriptions with Centers for Disease Control and Prevention (CDC) opioid data and drug-name field searches.

To visualize the relationship between opioid-prescribing volume and PDMP use, we divided clinicians into deciles by volume as measured by total opioid-days supplied. To summarize this relationship in tabular form, we aggregated the deciles into 3 groups: below median, above median to below top decile, and top decile. We also classified clinicians by whether they queried the PDMP. Within each subgroup, we assessed clinical specialization, opioid prescribing, and PDMP use.

This study was overseen by Columbia University's Institutional Review Board. We considered *P* values less than .05 from 2-sided tests to be statistically significant and conducted analyses using Stata/MP 17 (StataCorp).

## Results

There were 16 378 clinicians who prescribed opioids (Table 1). A slim majority were physicians and one-third were nurse practitioners or physician assistants. The average prescriber supplied 747 days of opioids to 31.9 distinct patients. While most opioid prescribers engaged with the PDMP, many did not: 4 in 10 did not search the PDMP during this period and 2 in 10 did not hold an account.

**Table 1.** Specialization, prescribing, and PDMP use among opioid prescribers.

	All opioid prescribers	By opioid-prescribing volume^a^			By use of search^a^	
		Below median	Median to top decile	Top decile	No searches	Had searches
Specialization, No. (%)						
Primary care physician	4673 (28.5)	1576 (19.3)	2058 (31.2)	1039 (63.5)	1323 (19.4)	3350 (35.1)
Specialist physician	4537 (27.7)	2630 (32.3)	1810 (27.5)	97 (5.9)	2566 (37.6)	1971 (20.6)
Nurse practitioner	2745 (16.8)	1231 (15.1)	1194 (18.1)	320 (19.5)	798 (11.7)	1947 (20.4)
Physician assistant	2404 (14.7)	1083 (13.3)	1154 (17.5)	167 (10.2)	657 (9.6)	1747 (18.3)
Opioid prescribing, mean ± SD						
Days supplied	747 ± 2858	29 ± 26	474 ± 407	5422 ± 7508	200 ± 765	1138 ± 3636
Fills dispensed	57.1 ± 131.4	8.8 ± 9.5	63.6 ± 63.5	270.9 ± 313.9	32.3 ± 77.8	74.7 ± 156.5
Distinct patients	31.9 ± 54.2	8.2 ± 9.2	46.9 ± 54.7	89.5 ± 99.7	24.6 ± 50.3	37.1 ± 56.2
PDMP searching						
Any search, No. (%)	9555 (58.3)	3454 (42.4)	4592 (69.7)	1509 (92.2)	0 (0.0)	9555 (100.0)
EMR-integrated,^b^ No. (%)	6995 (42.7)	2496 (30.6)	3353 (50.9)	1146 (70.0)	0 (0.0)	6995 (73.2)
No. of searches, mean ± SD	68.9 ± 301.5	17.2 ± 185.0	69.5 ± 249.3	324.3 ± 640.7	0.0 ± 0.0	118.1 ± 387.3
Has PDMP account, No. (%)	12 917 (78.9)	5812 (71.3)	5537 (84.1)	1568 (95.8)	3362 (49.3)	9555 (100.0)
No. of clinicians	16 378	8154	6587	1637	6823	9555

This table reports the characteristics of opioid prescribers overall, for subgroups defined by prescribing volume measured in days' supply, and for subgroups defined by whether the clinician searched the PDMP. Prescribing, searching, and account-holding were measured during the 180-day period starting February 1, 2023. Specialization derived from NPPES data. Specialization shares do not sum to 100% because specializations with small shares were omitted from the table. Primary care physicians are defined as those with specialization in Family Medicine, Internal Medicine, or General Practice. Searches that were conducted by a prescriber's PDMP delegates were attributed to that prescriber.

Abbreviations: EMR, electronic medical record; NPPES, National Plan and Provider Enumeration System; PDMP, prescription drug monitoring program; SD, standard deviation.

^a^Across all rows, all tests of equality of means across specified subgroups were statistically significant (*P* < .001).

^b^Prescribers with searches performed within an EMR system, which is possible when the EMR is integrated with the PDMP.

**Figure 1.**
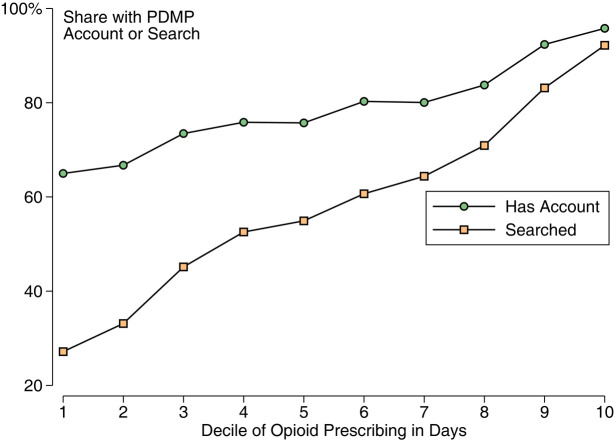
Rates of prescription drug monitoring program (PDMP) searching and account-holding by decile of opioid-prescribing volume. There were 16 378 clinicians who prescribed opioids during the study period. Prescribers are grouped into deciles according to their volume of opioid prescribing in total days supplied during the 180-day period starting February 1, 2023.

Prescription drug monitoring program use was more common among higher prescribers (Figure 1). Moving up 1 decile of opioid-prescribing volume was associated with a 6.7% point increase in the probability of searching (*P* < .001) and a 3.2% point increase in the probability of holding an account (*P* < .001). The vast majority of the highest-decile opioid prescribers searched the PDMP. Still, 7.8% of the highest-decile opioid prescribers did not search and 4.26% did not even have an account (Table 1).

Clinicians who did not search the PDMP were disproportionately specialist physicians and generally prescribed less (Table 1). Nonetheless, 32.2% of opioid fills and 11.1% of opioid days came from clinicians who never searched the PDMP.

## Discussion

Most clinicians are aware of the risks of opioid prescribing and prescribe opioids safely. Still, we found that a group of clinicians continues to prescribe opioids without using the tools available and required to ensure safe prescribing. The overrepresentation of low-volume prescribers in this group may reflect a lack of information about state mandates. That some of the highest-volume prescribers also do not use the PDMP, however, suggests that some prescribers believe that the mandate is not enforced and/or find the cost of engaging with the PDMP high relative to the benefits. Gaining access to and querying the PDMP may represent yet another time-consuming administrative burden for clinicians.^6-8^ Limitations of this study include the use of data from 1 state and lack of detailed patient data.

## Conclusion

Our analysis suggests that more work may be needed to engage clinicians in using PDMPs to assess patient opioid-prescribing history. Policy makers can leverage several evidence-based strategies to promote PDMP use based on 2 recent randomized trials. Integrating PDMPs with electronic medical records can increase query rates,^9^ and messaging clinicians about state requirements encourages account-holding and querying.^10^ These efforts could be paired with other work to reduce clinicians' administrative burdens and provide them with more time for high-value care activities. By addressing informational, administrative, and behavioral barriers, states and health care organizations can raise PDMP engagement and thereby encourage informed prescribing of opioids and other controlled substances.

## Supplementary Material

qxad067_Supplementary_Data
