# Correction to: Prescription drug monitoring program use by opioid prescribers: a cross-sectional study

**DOI:** 10.1093/haschl/qxae006

**Published:** 2024-03-04

This is a correction to: Adam Sacarny, Ian Williamson, Weston Merrick, Tatyana Avilova, Mireille Jacobson, Prescription drug monitoring program use by opioid prescribers: a cross-sectional study, *Health Affairs Scholar*, Volume 1, Issue 6, December 2023, qxad067, https://doi.org/10.1093/haschl/qxad067

In the originally published version of this manuscript, there were understated account-holding rates by prescribers. These errors did not affect any other results.

In the **Abstract**, the end of the third sentence should read: “[…]2 in 10 did not hold an account.” instead of: “[…]3 in 10 did not hold an account.”

In section **Results**, the last part of the final sentence of the first paragraph should read: “[…]2 in 10 did not hold an account.” instead of: “[…]3 in 10 did not hold an account.” In the second paragraph, end of the second sentence should read: “[…] and a 3.2% point increase in the probability of holding an account (*P* < .001).” instead of: “[…] and a 4.5% point increase in the probability of holding an account (*P* < .001).” The end of the fourth sentence should read: “[…]and 4.22% did not even have an account[…]” instead of: “[…]and 6.8% did not even have an account[…]”.

Data was errored in **Table 1** in the line “Has PDMP Account, No. (%)”. Figures along this line should read:

**Table:** 

12,917 (78.9)	5,812 (71.3)	5,537 (84.1)	1,568 (95.8)	3,362 (49.3)	9.555 (100.0)

instead of:

**Table:** 

11,300 (69.0)	4,756 (58.3)	5,018 (76.2)	1,526 (93.2)	1,745 (25.6)	9.555 (100.0)

**Figure 1** was errored: the share of clinicians with an account was understated, especially among low-volume opioid prescribers. **Figure 1** should read:

**Figure:**
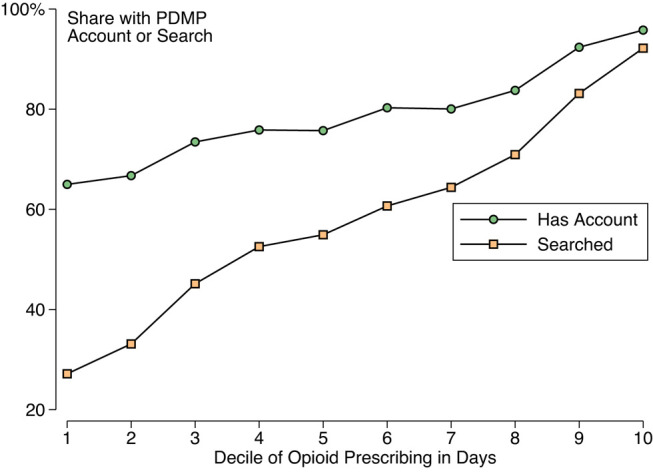


instead of:

**Figure:**
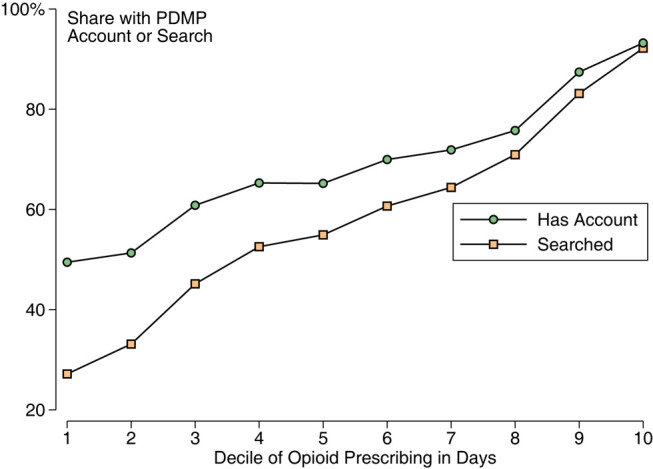


These errors have been emended in the article.

